# Nitrates and Nitrites in Vegetables and the Health Risk

**DOI:** 10.3390/foods14173037

**Published:** 2025-08-29

**Authors:** Ana Maria Dodocioiu, Gilda-Diana Buzatu, Mihai Botu

**Affiliations:** 1 Department of Horticulture & Food Science, Faculty of Horticulture, University of Craiova, 13 A.I. Cuza Street, 200585 Craiova, Romania; anadodocioiu@gmail.com (A.M.D.);; 2 Department of Biology and Environmental Engineering, Faculty of Horticulture, University of Craiova, 13 A.I. Cuza Street, 200585 Craiova, Romania

**Keywords:** nitrates, nitrites, vegetables, health risk assessment (HRI)

## Abstract

The research investigates nitrate and nitrite concentrations in vegetables sold at agri-food markets in Craiova, Dolj County. Vegetable samples were purchased from markets and sourced from the primary agricultural regions of Dolj County, ensuring a representative selection. A total of 300 samples were collected, with 20 samples taken from each of 15 vegetable species at commercial maturity. This research also aimed to estimate the contribution of each type of vegetable to the intake of nitrates/nitrites ingested through consumption, as well as to carry out an assessment of the risk to human health associated with the consumption of these vegetables. Our analysis showed that only three vegetables (tomatoes, eggplants, and bell peppers) exceeded the maximum permissible nitrate levels (MPL). The MPL for nitrite content was exceeded in several vegetables, including eggplant, green bean, lettuce, cabbage, dill, spinach, and lovage. For nitrates, the Hazard Risk Index (HRI) was consistently below 1 across all samples, with the sole exception of children’s consumption scenario. The HRI for nitrite was also below 1 for all samples, suggesting an absence of exposure risk. The findings from this study suggest that the consumption of vegetable products poses an insignificant risk in terms of nitrate and nitrite intake.

## 1. Introduction

Nitrates and nitrites, naturally occurring in vegetables, processed meats, and drinking water, have attracted considerable scientific attention due to their potential health effects. Nitrates are compounds that naturally exist in soil and water and can accumulate in plants, particularly under specific agricultural practices [1]. When consumed, nitrates can be converted to nitrites in the body, leading to potential health problems such as “blue baby syndrome” [2,3] and can be associated with different types of cancer [4,5,6]. Plants absorb nitrogen from the soil through the roots, mainly in the form of nitrates and less in the form of ammonium ions (${NH}_{4}^{+}$), as these are quickly oxidized in the soil to nitrates with the help of microorganisms. Once in the plant, nitrates are reduced to ammonium both in the roots (leguminous plants) and in the leaves (tomatoes, cucumbers, potatoes, etc.) under the action of nitrate reductase and nitrite reductase.

Vegetables are important sources of nitrates, especially green leafy vegetables such as spinach and lettuce, as well as root vegetables like beetroot. Agricultural practices, including the use of nitrogen fertilizers, contribute to high nitrate levels [7,8].

The occurrence of nitrates in vegetables, food and water has been a topic of scientific interest for many years. Fruit and vegetable consumption is the predominant source of dietary nitrate, significantly exceeding contributions from water and animal products [9,10]. While nitrate and nitrite intake has potential health implications, the benefits of consuming vegetables are well recognized, and the risks posed by nitrate from vegetables are considered minimal compared to those from processed meats [7,8,10]. Santamaria [7] highlights that although nitrate exposure through vegetables may raise concerns, their nutritional benefits outweigh the risks. Similarly, Luetic et al. [8] emphasize the importance of vegetable consumption despite nitrate content, and Erichsen et al. [10] point out that dietary nitrate sources from vegetables generally contribute less to health risks than processed meats.

Recent studies have reported wide variability in nitrate and nitrite levels across different types of vegetables and regions. Leafy vegetables, such as spinach and lettuce, typically contain the highest nitrate concentrations, often exceeding 2000–3000 mg/kg fresh weight in intensive farming systems [7,8]. Root vegetables, including beetroot and radishes, also show elevated values, while fruit vegetables like tomatoes and cucumbers tend to have lower concentrations, generally under 500 mg/kg. Geographical comparisons reveal significant differences, largely driven by environmental conditions, fertilization practices, and regulatory enforcement. For instance, vegetables grown in Northern and Central Europe such as Belgium, Germany, or the Netherlands tend to accumulate higher nitrate levels due to lower sunlight exposure and higher nitrogen input, compared to those cultivated in Southern Europe or Mediterranean countries [7,8,9,10]. In some cases, regional average levels have been found to exceed EU maximum permissible limits for certain crops. These differences highlight the importance of local assessments, particularly in countries like Romania where open-field vegetable production is widespread and climatic conditions are favorable for nitrate uptake.

Nitrate concentration can vary significantly due to several factors including plant species, growing conditions, seasonal variations, plant maturity, harvesting and storage and quality of water used for irrigation [7,8,11,12]. Studies have shown not only considerable differences in nitrate levels between different vegetables and foods, but also significant variability between samples of the same type of vegetable. This variability poses challenges for dietary guidelines and food safety regulations, making regular monitoring and assessment of nitrate levels important.

Understanding these factors is crucial for consumers, farmers, and policy makers to make informed decisions about agricultural practices, food safety, and dietary recommendations to minimize the health risks associated with high nitrate intake.

The study focuses on nitrates and nitrites in vegetables products from Dolj County, by including both vegetables with established maximum permissible nitrate/nitrite limits (regulated) and those without such limits (unregulated). It further assesses how these different vegetable types contribute to overall nitrate or nitrite intake of two population groups and evaluates the associated health risks.

Dolj County is located in the southern part of Romania and encompasses a total agricultural area of 588,945 hectares. Dolj County exhibits a diverse pedological structure that plays a significant role in agricultural land use and in the potential for nitrate accumulation in vegetable crops. The region includes eight principal soil classes with 16 dominant soil types. Chernozioms are the most widespread, occupying large portions of the Oltenia Plain and offering high natural fertility, making them particularly favorable for vegetable and cereal cultivation. In the northern hilly areas, Luvisols and Preluvosols are predominant; these soils are characterized by the presence of an argillic horizon that can limit water percolation and nutrient mobility. Aluviosols, present in the floodplains of the Jiu and Desnățui rivers, are fertile but susceptible to temporary waterlogging. Additionally, poorly drained zones across the county host Gleysols and Stagnosols, which are commonly associated with high groundwater levels and reduced oxygen availability in the root zone. In the southeastern part of the county, Psamosols sandy soils with inherently low nutrient retention are intensively cultivated using irrigation and controlled fertilization strategies. Less extensive but agriculturally relevant areas are covered by Vertosols, which are clay, rich and prone to swelling, shrinking behavior, and by saline and sodic soils such as Solonchaks and Solonetz, which can impose severe constraints on crop productivity. The heterogeneity of these soil types contributes to the spatial variability of nitrate accumulation in edible plant tissues and highlights the necessity of region-specific monitoring and risk assessment efforts. As a result, a significant portion of the vegetables grown in Dolj are not only consumed locally but are also distributed to other regions across the country. These products are widely available in local markets, especially in the main urban center of Craiova, where nine local markets are known for offering fresh, locally grown vegetables directly from producers.

In 2023, Dolj County ranked fourth nationally in total vegetable production. These factors, along with the intensive use of fertilizers in open-field vegetable farming, make Dolj County a relevant area for evaluating nitrate and nitrite accumulation in edible crops and assessing the associated dietary health risks. The region’s diversity in soil types and its high production volume provide a representative case for understanding nitrate variability and potential exposure in the population.

## 2. Results

The results presented in Table 1, Table 2 and Table 3 are based on the analysis of 300 vegetable samples belonging to 15 different species. Each species was represented by 20 samples, which were grouped into three categories: leafy vegetables, root and tuber vegetables, and fruit vegetables.

The nitrate concentration values of the selected fresh vegetables (including mean, median, concentration range, and variability (standard deviation) are expressed in mg/kg of fresh weight (FW). Nitrate concentrations for all analyzed vegetable products from the three groups were compared with the maximum permissible limits (MPLs) established by Order No. 1/2002 [13] in Romania. This national regulation was used as a reference because the current European legislation, Regulation (EU) 915/2023 [14], sets MPLs for nitrates only in a limited number of vegetables, namely spinach, lettuce, and arugula. Romania currently applies the EU framework; however, Order No. 1/2002 remains relevant for evaluating a broader range of vegetable products not specifically covered by EU limits. The results reveal significant variations in nitrate levels within the same vegetable species.

The mean nitrate content of the six fruit vegetables ranges from 53.2 mg/kg (cucumber) to 145.3 mg/kg (eggplant). Eggplant has the highest nitrate content among the studied vegetables followed by green bean, bell pepper, hot pepper, tomato and cucumber. It is evident from the results shown in Table 1 that eggplant, green bean and bell pepper show considerable variation in nitrate contents between different samples of the same vegetable. The analyzed tomato samples exceeded the MPL, which is 150 mg/kg, only for 10% of the analyzed samples.

The cucumber samples analyzed had values below MPL, which is 200 mg/kg.

For the eggplant samples, MPL was exceeded, which is 300 mg/kg, in 10% of the analyzed samples, with the recorded concentration varying between 45.0 and 870.0 mg/kg.

The high standard deviation (SD) values observed in some vegetable species, such as eggplant (145.3 ± 179.9 mg/kg NO_3_^−^), green bean (122.9 ± 70.6 mg/kg NO_3_^−^), and bell pepper (96.4 ± 82.5 mg/kg NO_3_^−^), reflect the inherent variability in nitrate accumulation within and between samples collected from different local producers and agricultural subregions of Dolj County, Romania. This variability is not random but reflects the following site-specific and crop-specific factors. Vegetable samples with extremely high nitrate concentrations were associated with producers from the left of the river Jiu area, where psamosols are found on an area of 74,000 ha, with a high coarse sand content of 67.1–78.2 (2–0.2 mm), moderate and weakly acidic pH (5.64–6.15) and low humus content (0.64–0.92%), determining the need to apply high amounts of nitrogen fertilizers, especially ammonium nitrate, due to its high nitrogen content, low cost, and availability. Psamosols favor the leaching of nitrogen in the form of nitrates, but, paradoxically, in the absence of adequate fertilization management, they can also lead to excessive accumulations of nitrates in plants.

Nitrogen fertilization, applied in high doses or at inappropriate times, has an increased risk of leading to nitrate accumulations in plant tissues. This phenomenon is accentuated on sandy soils due to the rapid availability of nitrogen in the form of nitrates, as a result of accelerated mineralization and increased mobility of this ion in the soil profile. These operational and environmental inconsistencies explain the SD value of 82.5 mg/kg across samples.

For the samples of green bean, the recorded concentrations varied within wide limits, recording values between 50.0 and 320.0 mg/kg; for this vegetable product, the legislation in force does not establish the maximum permissible limit.

For the bell pepper samples analyzed, the nitrate concentration recorded values between 24.0 and 420.0 mg/kg, exceeding the MPL of 150 mg/kg in 5% of the analyzed samples.

For the hot pepper samples, all the recorded values were below the MPL of 150 mg/kg.

The amounts of nitrates in fruit vegetables are lower than those in leafy vegetables due to the fact that, in fruit vegetables, assimilation products circulate, resulting from the photosynthesis process carried out at the leaf level.

Within the fruit vegetable category, the nitrate concentration in descending order is as follows: eggplant > green bean > bell pepper > hot pepper > tomato > cucumbers.

The mean nitrate content of the six leafy vegetables ranges from 157.2 mg/kg (cabbage) to 794.35 mg/kg (lovage) (Table 2). None of the leafy vegetable samples analyzed exceeded the MPL.

Especially in leafy vegetables, nitrate tends to accumulate significantly in the leaves due to the physiological processes involved in their growth and development. This accumulation is influenced by several factors, including the plant’s growth stage, environmental conditions, and nitrogen availability in the soil. Leafy vegetables such as spinach, lettuce, and parsley are known for their high nitrate content [12].

Within the leafy vegetable category, the nitrate concentration, expressed in mg/kg fresh weight (FW), in descending order is as follows: lovage > lettuce > dill > spinach > parsley > cabbage.

Regarding the nitrate content recorded in root and tuber vegetables (Table 3), no exceedances of the maximum permissible limits accepted by the legislation were recorded.

The highest nitrate concentration was recorded in one potato sample (210 mg/kg), which represents 5% of the samples analyzed for this species (*n* = 20).

Within the root and tuber vegetable category, the nitrate concentration in descending order is as follows: potatoes > carrots > onions.

The nitrite concentration values of the selected fresh vegetables (mean, median, concentration range and variability (SD)) expressed as mg/kg of fresh weight are presented in Table 4, Table 5 and Table 6. The nitrite concentration values in all analyzed vegetable products grouped into the three categories were compared with the MPL for nitrites established by Order No. 1/2002 in Romania, since these limits have not been established at the European level.

Regarding nitrite content in fruit vegetables, eggplant exhibited the highest mean concentration (0.78 mg/kg) and the widest variation (0.40–1.90 mg/kg), indicating a tendency for higher nitrite levels in some samples, correlated with nitrate content in the same samples. Nitrites result from the enzymatic reduction of nitrates, and the observed differences can be attributed to soil factors and variable fertilization practices among producers, as well as to the physiological characteristics of eggplant, which may influence nitrogen metabolism depending on the cultivation conditions.

Other vegetables have similar mean levels around 0.49 mg/kg (tomato, bell pepper, hot pepper) to 0.54 mg/kg (cucumber), with the range mostly between 0.20 mg/kg (tomato, green bean, bell pepper, hot pepper) and 0.80 mg/kg (cucumber, bell pepper, hot pepper).

All analyzed fruit vegetables have mean concentrations well below the maximum permissible level (1 mg/kg). Despite average concentrations being below the established limit, the analysis of maximum values shows that the highest nitrite levels were found in eggplants (1.90 mg/kg) and green beans (1.20 mg/kg), both exceeding the maximum permissible limit (MPL). Although certain samples (15% of the eggplant samples and 10% of the green bean samples) exceeded the maximum permissible level (MPL), potential health risks depend on the actual daily intake relative to the acceptable daily intake (ADI). Therefore, exceeding the MPL alone does not necessarily indicate a direct health risk, unless the ingested amount results in an estimated daily intake (EDI) that exceeds the ADI (i.e., HRI > 1).

Regarding the mean ${NO}_{2}^{-}$ content of leafy vegetables, it varied between 0.57 mg/kg (parsley) and 0.94 mg/kg (spinach).

If we refer to the mean nitrite value, all leafy vegetables have nitrite concentrations below the maximum permissible limit. Nitrite concentration range in leafy vegetables has values between 0.10 mg/kg (cabbage) and 1.70 mg/kg (lettuce). From the leafy category, only in the parsley samples was the maximum permissible limit not exceeded; all values for this vegetable were below 1 mg/kg. Lettuce and spinach, because of their higher average levels, require careful monitoring, as some samples may approach or slightly exceed the MPL. Exceedances of nitrites were recorded in 40% of lettuce samples, 25% of cabbage and spinach samples and 5% of dill and lovage samples.

Regarding the nitrite content recorded in root and tuber vegetables (Table 6), no exceedances of the maximum permissible limits accepted by the legislation were recorded.

The nitrite mean concentration for root and tuber vegetables does not show variations, with values of 0.60 mg/kg (carrots, onions) to 0.65 mg/kg (potatoes) being below 1 mg/kg, consistent with other research [15].

The nitrite concentrations in carrots, potatoes, and onions range from 0.30 mg/kg (carrots, onions) to 0.90 mg/kg (carrots, potatoes, onions), showing some variability but within a relatively narrow range. While the nitrite concentrations were below the MPL, an acceptable safety level can only be confirmed when the estimated intake remains below the ADI, as reflected by a Hazard Risk Index (HRI) lower than 1. However, some maximum values (0.90 mg/kg) are close to the limit, highlighting the importance of monitoring and controlling cultivation and harvesting processes to prevent exceeding safety thresholds. Overall, root and tuber vegetables do not pose a health risk based on their nitrite content, but maintaining proper agricultural practices is recommended to prevent elevated levels of nitrites.

**Figure 1:**
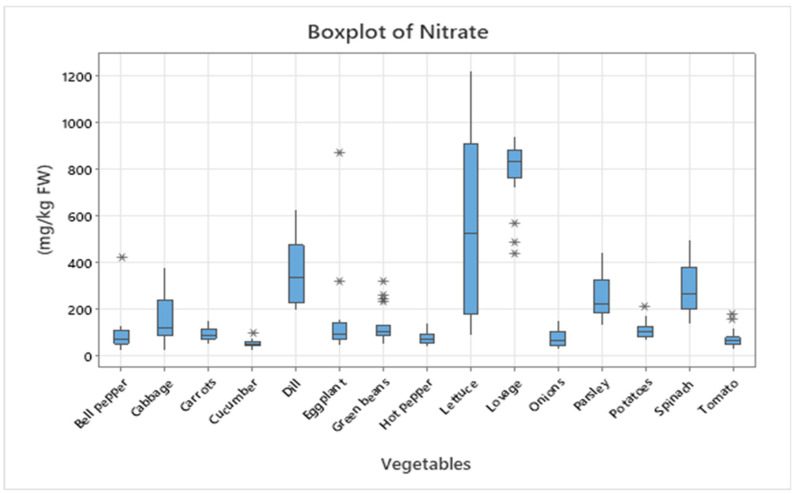
Distribution of nitrate concentrations (mg/kg FW) in 15 vegetable species analyzed from Dolj County. Boxplots display median, interquartile range, minimum and maximum values, and outliers.

**Figure 2:**
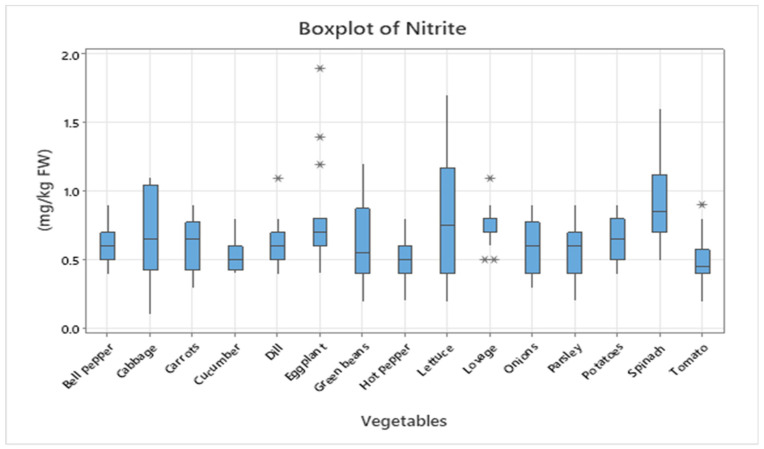
Distribution of nitrite concentrations (mg/kg FW) in 15 vegetable species analyzed from Dolj County. Boxplots display median, interquartile range, minimum and maximum values, and outliers.

**Figure 3:**
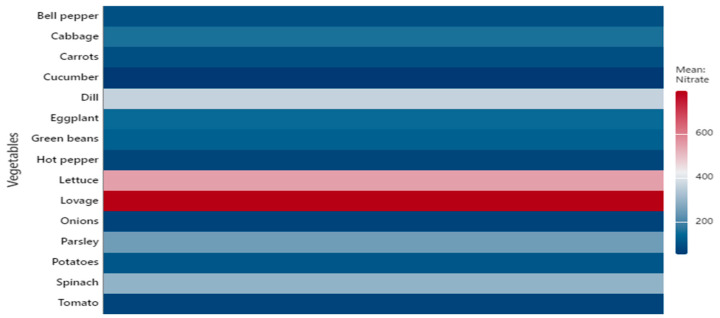
Heatmap of mean nitrate concentrations (mg/kg FW) in 15 vegetable species. Color scale represents average values, highlighting higher accumulation in leafy vegetables compared to fruit and root/tuber crops.

**Figure 4:**
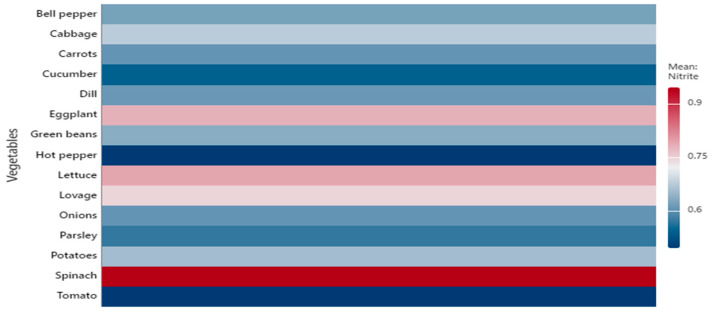
Heatmap of mean nitrite concentrations (mg/kg FW) in 15 vegetable species. Color scale represents average values, showing a more uniform distribution compared to nitrate levels.

Leafy vegetables generally displayed higher nitrate concentrations than fruit or root and tuber vegetables, consistent with previous findings [7,8,10]. To illustrate the variability and distribution of concentrations, the data are presented as boxplots (Figure 1 and Figure 2). In addition, heatmaps (Figure 3 and Figure 4) were generated based on mean values, providing a concise visual representation of the differences among species.

The boxplot in Figure 1 illustrates the distribution of nitrate concentrations (mg/kg FW) in 15 vegetable species analyzed. The highest median values were recorded in lovage and lettuce, with concentrations ranging between approximately 800 and 850 mg/kg FW and maximum values exceeding 1200 mg/kg FW. Moderate levels (200–400 mg/kg FW) were observed in dill, spinach, parsley, and cabbage, while the lowest values (<100 mg/kg FW) were found in cucumbers, tomatoes, hot peppers, and onions. The high variability and presence of extreme values (outliers) in certain species, such as eggplant and dill, suggest significant influences of pedoclimatic conditions and fertilization practices on nitrate accumulation.

Figure 2 presents the boxplot illustrating the distribution of nitrite concentrations (mg/kg FW) in 15 vegetable species. Median levels generally range between 0.5 and 1.0 mg/kg FW, with isolated maximum values reaching or exceeding 1.5–1.9 mg/kg FW in eggplant, spinach, and lettuce. The highest medians were recorded in cabbage, spinach, and lettuce, while bell pepper, tomatoes, cucumbers, and parsley showed lower median concentrations (<0.6 mg/kg FW). The presence of outliers indicates significant differences between samples, highlighting the need for continuous monitoring of these compounds throughout the food chain.

The heatmap shows the mean nitrate concentrations (mg/kg FW) for the 15 vegetable species analyzed. The highest mean concentrations were recorded in lovage and lettuce, followed by dill, spinach, and parsley, consistent with the trends observed in the boxplot. Leafy vegetables generally exhibited significantly higher mean values than fruit or root vegetables, while cucumbers, hot peppers, tomatoes, and onions had the lowest means (Figure 3).

The heatmap of mean nitrite concentrations (mg/kg FW) shows a more uniform distribution among species compared with nitrates, with values generally ranging between 0.6 and 0.9 mg/kg FW. The highest means were observed in spinach, cabbage, lettuce, and eggplant, while hot peppers and cucumbers showed the lowest values. This representation clearly highlights the average-level differences among species and complements the boxplot analysis, which illustrates within-species variability (Figure 4).

In Table 7, we have summarised the health risk exposure to nitrate and nitrite through average consumption of vegetables for two population groups (children and adults), as recommended by the EFSA [16] based on our own calculation using data regarding the consumption availability of the population in 2022, published in 2024 by National Institute of Statistics [17] (for the data underlying these calculations see Table 13).

Nitrate and nitrite daily intake could only be calculated for the vegetable products tomato, cucumber, green bean, bell pepper, cabbage, potatoes and onions, since only for these the annual consumption in kg/year was made available by the NIS [17] for 2022; for the rest of the vegetable products, data were not found. These data represent nationally aggregated food availability per capita and offer the advantage of being systematically collected, standardized, and comparable across time.

Although these data include potential overestimations due to food waste, inedible parts, or losses in the supply chain, they remain the most comprehensive and up-to-date source available at the national level. In the absence of recent, detailed individual dietary intake data, particularly disaggregated by age, this approach provides a robust and consistent basis for estimating population-level exposure and supporting risk assessment efforts.

Furthermore, using a uniform average value for both adults and children ensures methodological consistency across the assessment, and while it may introduce some degree of uncertainty in child-specific estimates, it still allows for a comparative and conservative evaluation of potential risks. This approach is commonly accepted in preliminary exposure assessments where more granular data are unavailable.

Daily nitrate intake varied for the seven vegetable products listed above between 3.51 mg/kg (onions) and 29.10 mg/kg for potatoes.

Potato is the most consumed vegetable in Romania according to the data in Table 13 with, the Romanian population consuming 97.7 (kg/inhabitant)/year [17].

Calculating the daily intake for the highest nitrate concentration recorded in potatoes (210 mg NO_3_^−^/kg), we obtain a value of 56.21 mg/day. This value is approximately 93% higher than the intake calculated using the average nitrate content in potatoes (29.1 mg/day). Both values remain below the ADI of 3.7 mg/kg bw/day, corresponding to 222 mg/day for a 60 kg adult and about 100 mg/day for a 27 kg child, as established by EFSA.

In the 300 vegetable samples analyzed during 2023, EDI ranged between 0.48 mg/kg body weight/day (cucumber) and 7.23 mg/kg bw/day (lovage) for children and between 0.21 mg/kg bw/day (cucumber) and 3.25 mg/kg bw/day (lovage) for adults. For adults, the estimated daily intake (EDI) values do not exceed the acceptable daily intake (ADI) of 3.7 mg/kg bw/day. However, for children, the EDI exceeds the ADI for lettuce (4.99 mg/kg bw/day) and lovage (7.23 mg/kg bw/day). Although the average nitrate concentrations in these two vegetables exceed the MPL, the health risk to children depends on the amount consumed and whether the estimated intake exceeds the ADI. For the children’s consumption scenario, the vegetable product lovage recorded an HRI > 1, indicating a potential non-carcinogenic health risk and suggesting the need for moderation in consumption.

Daily nitrite intake for the analyzed vegetables had values below 0.01 mg/day (green bean) to 0.08 mg/day (cabbage). Although for each analyzed product, the daily nitrite intake values were lower than the ADI value of 4.2 mg ${NO}_{2}^{-}$/day for adults and 1.89 mg ${NO}_{2}^{-}$/day for children [18], the calculated ADI for each vegetable product varied between 5.71% (26.6% of the samples) and 11.42% (6.6% of the samples for children), and for adults between 2.85% (53.3% of the samples) and 5.71% (for 13.3% of the samples).

The nitrite values recorded for EDI varied between 0.004 mg/kg body weight/day (26.6% of samples) and 0.008 mg/kg bw/day/day (6.6% of samples) in children, and in adults, it varied between 0.002 mg/kg bw/day (73.3% of samples) and 0.003 mg/kg bw/day (26.7% of samples). In these conditions, we recorded HRI values much lower than 1, indicating that there is no danger to human health in terms of ingested nitrite.

In Table 8, we summarize the health risk exposure to nitrate and nitrite through vegetable consumption for the average intake of two population groups (children and adults). According to FAO/WHO recommendations (2002), the total daily intake of fruits and vegetables combined should be at least 400 g/day [19]. In this study, this reference value was applied to assess potential nitrate/nitrite exposure through vegetable consumption alone, which may result in a conservative estimation.

Due to the lack of specific FAO/WHO recommendations for children, the 400 g/day value was applied uniformly for both adults and children, which may lead to an overestimation of nitrate or nitrite exposure in the pediatric population. While the 400 g/day intake scenario allows comparison across vegetable types, it may not reflect realistic daily intakes for all vegetables (e.g., lovage or dill, which are typically consumed in small quantities). Therefore, the results should be interpreted as a worst-case exposure estimate.

For children, leafy vegetables like lettuce (8.11 mg/kg bw/day) and lovage (11.76 mg/kg bw/day) show the highest nitrate EDIs, indicating higher potential intake levels. For adults, these values are proportionally lower but follow the same trend.

It is observed that the values exceed the ADI only in the case of children for lettuce (219.18%), dill (145.67%), and spinach (117.83%). For lovage, high values are recorded for both children and adults, significantly exceeding the ADI (317.83% in children and 142.97% in adults). The consumption of these leafy vegetable products that surpass the ADI indicates a potential health concern for frequent consumers. In particular, the HRI values for children consuming lettuce and lovage exceed 1 (1.14 and 1.65, respectively), clearly indicating a potential non-carcinogenic health risk associated with regular consumption. This finding highlights the need for consumption moderation, especially among vulnerable groups such as children. For adults, the HRI values remain below 1, suggesting no immediate risk, although high EDIs in some leafy vegetables like lovage warrant attention.

Within the category of leafy vegetables, only two products have an HRI greater than 1, namely lettuce (1.14) and lovage (1.65), in the children scenario, indicating a potential non-carcinogenic health risk.

Leafy vegetables, especially lettuce and lovage, contribute significantly to nitrate exposure, with some values exceeding safety thresholds, especially for children. Continuous monitoring and consumption moderation are advised for high nitrate vegetables to mitigate potential health risks, particularly for vulnerable groups like children.

Regarding nitrite exposure, leafy vegetables again show higher nitrite EDIs, with lettuce (0.011 mg/kg bw/day children, 0.005 mg/kg bw/day adults) and lovage (0.010 mg/kg bw/day children, 0.004 mg/kg bw/day adults) being notable.

The ADI values are well below 100%, indicating lower risk levels compared to nitrates. HRIs are below 1, suggesting minimal immediate risk from nitrite at current levels. Nitrite levels are generally within acceptable limits across all vegetables.

## 3. Discussion

This study aims to determine the presence of nitrates and nitrites in vegetable products from agri-food markets in Dolj County. The analyzed vegetables encompass a range of types, including tomato, cucumber, eggplant, green bean, bell pepper, hot peppers, carrots, potatoes, onions, dill, parsley, lovage, lettuce, cabbage, and spinach. For analysis, these were categorized into three groups: fruit, leafy, and root tuber vegetables.

The study also evaluates the impact of vegetable consumption on nitrate and nitrite intake in two population groups (children and adults) and assesses the potential health risks associated with nitrate and nitrite exposure based on daily nitrate/nitrite intake (EDI, ADI, HRI).

The nitrate and nitrite levels observed in this study varied significantly among vegetable species, consistent with previous findings that leafy vegetables (e.g., lettuce, spinach, parsley) tend to accumulate higher levels of nitrates compared to fruit vegetables (e.g., tomatoes, peppers) or root vegetables (e.g., potatoes, carrots) due to their higher transpiration rates and nitrogen metabolism efficiency [7].

Although direct data on planting or fertilization methods were not collected from each producer, most of the vegetables sold in the Craiova markets originate from small- to medium-sized farms using open-field cultivation. This method exposes crops to natural variations in sunlight and temperature, factors known to influence nitrate accumulation with lower light intensity often associated with increased nitrate content [16].

Moreover, agronomic practices specific to Dolj County commonly involve the frequent and often excessive application of nitrogen-based fertilizers, particularly ammonium nitrate. When interacting with certain soil types, such practices may significantly contribute to the elevated nitrate concentrations observed in specific vegetable groups. These combined factors may account for the species and site-specific variations in nitrate and nitrite content documented in this study.

Although the present study provides relevant data on nitrate and nitrite levels in commonly consumed vegetables in Dolj County, it represents primarily a preliminary assessment of the factors influencing the accumulation of these compounds. While species-specific differences have been identified, a more comprehensive investigation into the complex interactions among multiple agronomic and environmental variables is necessary to fully elucidate the variability in nitrate and nitrite content.

Numerous studies have shown that nitrate accumulation in vegetables is not determined solely by plant species, but is also affected by several interacting factors such as soil type and pH, fertilization regime (especially nitrogen-based fertilizers), irrigation frequency, climatic conditions (e.g., solar radiation, temperature, precipitation), as well as cultivation methods (e.g., open field vs. greenhouse, conventional vs. organic systems). These variables can act synergistically, influencing the nitrate uptake, assimilation, and storage pathways within plant tissues.

A comprehensive analysis of these interactions, for example, through multivariate statistical approaches (e.g., principal component analysis, regression models), would allow for a more integrated understanding of the determinants of nitrate and nitrite levels in vegetables. Future research should aim to include such analyses, along with detailed records of cultivation conditions and fertilization practices, in order to identify the key contributors to excessive nitrate or nitrite accumulation and to propose effective mitigation strategies.

The observed differences in nitrate and nitrite content among the various vegetable types can be attributed to intrinsic physiological and biochemical characteristics of the plants, as well as environmental and agronomic factors. Leafy vegetables typically exhibit higher nitrate accumulation due to their active nitrate uptake through roots and subsequent translocation and storage in leaf tissues via the xylem transpiration stream. This is consistent with the role of nitrates as primary nitrogen sources for plant metabolism and growth. In contrast, root and fruit vegetables tend to accumulate lower nitrate levels because nitrates are either metabolized more rapidly or partitioned differently within plant organs.

Additionally, nitrite accumulation is generally lower than nitrate levels due to the rapid enzymatic conversion of nitrites in plant tissues. However, environmental stressors, such as low light intensity or excess nitrogen fertilization, may inhibit nitrite reductase activity, leading to transient increases in nitrite content.

From a health risk perspective, these biochemical and physiological differences significantly affect the estimation of dietary exposure to nitrates and nitrites. Vegetables with higher nitrate content, especially leafy greens, may pose a greater risk of exceeding acceptable daily intake (ADI) limits, particularly in sensitive populations such as children. Therefore, the variability in nitrate and nitrite levels must be carefully integrated into risk assessment models to provide accurate estimations of potential adverse health outcomes.

Our study’s evaluation of Estimated Daily Intake (EDI), Acceptable Daily Intake (ADI), and Health Risk Index (HRI) reflects these considerations by stratifying exposure based on vegetable type and consumption patterns. This approach highlights the importance of targeted dietary recommendations and regulatory monitoring to minimize nitrate and nitrite-related health risks.

The observed discrepancies in nitrate and nitrite content across different vegetables, compared to values reported in other studies, may be attributed to several agronomic and environmental factors that differ significantly between cultivation regions. Soil nitrate levels, influenced by both natural fertility and fertilization practices, play a crucial role in nitrate accumulation in plant tissues. In Dolj County, vegetable production takes place on a wide range of soil types. These soils differ considerably in structure, drainage, and natural nutrient content, which can affect nitrate dynamics in the root zone. Moreover, local farmers predominantly use ammonium nitrate as a nitrogen fertilizer, as it is one of the cheapest and most accessible nitrogen sources. This practice is a key factor contributing to elevated nitrate concentrations in vegetables. Furthermore, many small-scale farmers in the region do not calculate or adjust the applied fertilizer doses based on soil testing or crop needs, often applying excessive amounts of nitrogen fertilizers. These agronomic behaviors, combined with the diverse soil and climatic conditions in Dolj County, are likely to explain part of the variability observed in nitrate and nitrite concentrations in the current study. Therefore, integrating these environmental and agricultural determinants is essential for interpreting the findings and for developing context-specific risk mitigation strategies.

### 3.1. Nitrate Content

The nitrate accumulation in vegetables is a dynamic process contingent upon the synergistic effects of the plant’s genetic predisposition and the prevailing environmental conditions during its cultivation. Nevertheless, legislative instruments stipulate definitive upper limits for nitrate content in particular vegetable products for public health protection.

This highlights the inherent variability in nitrate levels within a given vegetable, demonstrating that there is no single fixed value but rather a range of concentrations dictated by the interplay of these influencing factors.

The variability observed in nitrate concentrations, particularly among leafy vegetables, may be attributed to a combination of agricultural and environmental factors. High levels exceeding the maximum permissible limits (MPLs) in certain samples, such as one eggplant (870.0 mg/kg) and several leafy vegetables, can be linked to excessive nitrogen fertilization, especially the use of synthetic fertilizers rich in nitrate and ammonium ions. Additionally, the soil type and pH significantly influence nitrate uptake; for instance, acidic or poorly aerated soils can enhance nitrate mobility and plant absorption.

The soils in Dolj County, notably chernozem and alluvial types, are fertile and generally neutral to slightly alkaline. However, localized areas may deviate in pH due to previous fertilization or irrigation practices, potentially facilitating greater nitrate accumulation. Moreover, vegetable cultivation, which is frequent for leafy vegetables, tends to use higher nitrogen inputs to maximize yields, contributing to elevated nitrate content.

Consequently, the values obtained in our study align with those reported in some other studies. However, a review of the literature reveals a range of reported values, with some studies indicating both higher and lower levels than those we determined.

Nitrate levels in vegetables vary not only between countries, but also between regions within the same country, as their accumulation in plants depends on several factors such as soil type, pH, fertilization practices, irrigation management, climatic conditions and plant species.

The ${NO}_{3}^{-}$ concentration found in tomatoes is similar to values reported by other studies [20,21,22,23,24,25].

Several studies have reported nitrate concentrations in vegetables comparable to those found in our study. For example, similar values were observed for cucumbers [26,27,28,29], eggplants [30], lettuce, spinach, and parsley [26,29,31,32,33], as well as spinach and cabbage [28,29]. Lettuce nitrate values were particularly close to those reported by Dezganhah and Brkić [31,34].

Conversely, higher nitrate concentrations than those found in this study were reported for dill and parsley [29], as well as for tomato, cucumber, eggplant, green bean, lettuce, bell pepper, cabbage, spinach, parsley, dill, carrots, potatoes, and onions across various international studies [21,22,23,24,25,26,27,28,29,30,31,32,33,34,35,36,37,38,39,40,41,42,43,44,45,46,47,48,49,50,51,52,53,54,55,56,57]. On the other hand, some authors observed lower nitrate levels for these vegetables, especially tomato, cucumber, eggplant, and bell pepper [24,34,37,50,53,56], or even notably low values such as 6.4 mg/kg nitrate in tomatoes reported by Kmecl et al. [37], compared to 74.2 mg/kg in our study.

Our findings are consistent with those reported in regional studies, such as Kmecl et al. [37], which highlighted that vegetables grown under intensive fertilization regimes, particularly in protected environments and on nitrate-retentive soils, showed a higher tendency to exceed MPLs. In contrast, field-grown crops under balanced fertilization programs presented significantly lower nitrate levels.

Therefore, in addition to genetic and physiological traits of the species, the cultivation method, fertilization intensity, and edaphic factors (e.g., soil pH, organic matter content) must be considered as significant contributors to the differences observed in nitrate accumulation across vegetable species.

Potatoes and onions also showed values in line with other literature [22,23,29,44,48], although one study [24] reported potato nitrate levels up to three times higher than ours. Schuddeboom [58] noted lower nitrate levels in potatoes from Germany, Denmark, and Norway (60–93 mg/kg), which remain below the average found in our samples (108.8 mg/kg).

The high variability observed among studies is explained by factors such as soil and climate conditions, fertilizer application and irrigation practices. Moreover, leafy vegetables remain the most efficient nitrate accumulators due to their physiology and nitrate transport mechanisms through the xylem [7,8,29,31,34,35,36,37,38,39,40,41,42,43].

Table 9 presents the classification of vegetable products according to nitrate content, as proposed by Santamaria [7], a framework widely cited for its structured grouping of vegetables (low to very high nitrate classes). In this study, Santamaria’s system was applied only as a complementary reference to contextualize the Romanian data and enable comparisons with earlier literature, while quantitative exposure estimations were based on the latest EFSA recommendations (2020–2023). Accordingly, Table 10 shows the classification of the vegetables analyzed in this study following Santamaria’s categories, providing a benchmark for interpreting the results alongside contemporary EFSA-based dietary exposure and health risk calculations.

Table 10 presents the classification of the vegetable products studied according to their nitrate content into one of the five classes after Santamaria [7] (class I, very low < 200 mg/kg; class II, low 200–500 mg/kg; class III, middle 500–1000 mg/kg; class IV, high 1000–2500 mg/kg; class V, very high > 2500 mg/kg). This classification by class was made for all 300 samples, taking into account the fact that 20 samples were analyzed for each species.

The table analysis revealed that in the fruit vegetable category, consisting of tomatoes, cucumbers, and hot peppers, all 60 samples analyzed were under 200 mg/kg, which is considered to be the first class.

For eggplant, beans and bell peppers, the nitrate content has a higher variability. In total, 54 samples analyzed (representing 90%) were included in class I, 5 samples in class II, and 1 sample from eggplant in class 3, having a value of 870.0 mg/kg. In conclusion, from this category where 120 samples were analyzed, 95.01% were included in class I, 4.16% in class II, and 0.83% in class III.

Regarding the leafy vegetable category, from which we analyzed lettuce, cabbage, spinach, dill, parsley and lovage, with a total of 120 samples, it is noted that the nitrate content variation is much higher than in the fruit vegetable category, with a higher number of samples falling in class II, III and IV. Thus, 29.16% of the leafy vegetable samples are in class I, 43.33% in class II and 25.83% in class III and 1.67% in class IV.

For root and tuber vegetables (carrots, potatoes, onions), a total of 60 samples were analyzed. Of these, 98.33% fall into class I, containing nitrates below 200 mg/kg, and only one sample, in potatoes, which represents 1.67%, falls into class II, registering a value of 210 mg/kg.

Of the 300 vegetables samples analyzed, 69.34% are in class I, 19.33% in class II, 10.66% in class III and 0.67% class IV. In this study, no vegetable products were identified with a nitrate concentration higher than 1223 mg/kg, so there were no vegetable samples in class V.

The results presented regarding the nitrate content of the analyzed vegetable products confirm the classification in Table 10 and highlight the fact that the nature of the vegetable product is a decisive factor in the accumulation of nitrates.

This table indicates that a selective monitoring of vegetable products is necessary in the future, from which vegetable products with a high degree of accumulation should be analyzed in particular, i.e., the high (1000–2500 mg/kg) and very high (>2500 mg/kg) class.

Findings highlight the need for balanced vegetable consumption, considering both nutritional value and nitrate content, and monitoring species with higher accumulation.

In addition to the classification by Santamaria [7], the system proposed by Shen [15] provides a contamination-based interpretation, correlating nitrate levels with dietary recommendations. This approach offers a practical evaluation of health risk depending on how vegetables are consumed (raw, cooked, pickled), and is therefore useful for public health guidance. The classification is presented in Table 11.

This system categorizes nitrate content into four levels (I–IV), each associated with specific dietary recommendations (e.g., whether vegetables can be safely consumed raw, pickled, or only cooked), thus providing a more practical interpretation of nitrate exposure in terms of consumer health.

Based on this model, the vegetable samples analyzed in the present study were distributed into the corresponding contamination levels, as shown in Table 12. Out of the 120 fruit vegetable samples, 119 (99.17%) fell into level I (≤432 mg/kg), while 1 eggplant sample (0.83%) was classified in level III (870 mg/kg). Across the total of 300 samples, 39.67% fell into level I and only 0.33% into level III. No samples exceeded the threshold for levels IV or higher. This classification highlights that the vast majority of the vegetables analyzed can be considered safe for raw or cooked consumption under current dietary standards.

Regarding the leafy vegetable category, there is a higher variability of nitrate concentration in the six plant products included in this category, with values between 25 mg/kg (cabbage) and 1223 mg/kg (lettuce grown in the field). Of the 120 samples analyzed, 78 samples fall in level 1 (65%) that are slightly contaminated and can be eaten raw, 19 samples that are moderately contaminated and cannot be eaten raw, but can be pickled and cooked, fall in level II (15.83%) and 23 samples fall in level III (19.17%) and are heavily contaminated, and cannot be eaten raw or pickled, but can be cooked.

Leafy vegetables represent 40% of the total samples analyzed (*n* = 300), and of these, 26% fall in level I, 6.33% in level II and 7.67% in level III.

Regarding root and tuber vegetables, all 60 samples analyzed can be categorized, depending on nitrate concentration, as level I (100%). Referring to the total number of vegetable products studied, root and tuber vegetables are classified as level I and represent 20%.

Of the total of 300 samples analyzed, 85.67% fall in level I, and can be eaten fresh, 6.33% fall in level II, can be consumed both fresh, pickled and cooked, and 8% fall in level III and cannot be eaten raw or pickled, but can be cooked.

It is important to mention that certain analyzed vegetables are consumed partially or totally prepared thermally, and the specialized literature indicates that thermal treatments can lead to an increase or decrease in nitrate content. However, since the determinations were made on fresh and raw samples, and the consumption of these vegetables occurs predominantly in this form, the effect of thermal processing was not included in our analysis.

### 3.2. Nitrite Content

Concerning nitrites, as with nitrates, a substantial degree of variability exists in their concentrations within vegetables. Although some studies present values similar to those in our investigation, a review of the specialized literature reveals studies reporting both higher and lower nitrite values than those we determined.

Our findings for nitrite content in cucumber and eggplant samples align with the values reported in other studies [51].

Some authors report values much lower than those in the present study for tomato [21,37,49,51,53], cucumber [21,37,49,53], eggplant [53], green bean [37,59], pepper [37], lettuce [51,60], cabbage [8,21,51,53], spinach [51,53,59], carrots [37,51,53,59], potatoes [37,51], dill, parsley and onions [51].

In contrast to the results presented in this paper, there are also studies that report higher nitrite values in vegetables. Thus, higher values have been reported for cucumber [29,50], eggplant [21,29,50], green bean [50], pepper [29,50], lettuce [8,29,50,53,61], cabbage [29,50,62], spinach [8,29,50,61,63], parsley [29,50], carrots [29,50] and potatoes [21,29] and onions [29,50].

The nitrate and nitrite levels observed in this study varied significantly among vegetable species, consistent with previous findings that leafy vegetables (e.g., lettuce, spinach, parsley) tend to accumulate higher levels compared to fruit or root vegetables due to their nitrogen metabolism efficiency [64]. Similar results were reported in [65], which demonstrated that spinach and chard cultivated under greenhouse conditions showed enhanced nitrate uptake and distribution under optimal fertilization. Furthermore, [66] highlighted that baby foods and infant formulas containing vegetable derivatives also show variable nitrate concentrations, underlining the importance of monitoring across multiple dietary sources. In addition, [67] showed in a systematic review that dietary nitrate exposure remains an important risk factor for colorectal cancer, especially in populations consuming large amounts of leafy greens, while [68] confirmed the health risks from trace elements and nitrate contamination in infant formulas. These studies reinforce our conclusion that targeted monitoring and tailored dietary recommendations are necessary for sensitive groups such as children.

### 3.3. Potential Changes in Nitrate and Nitrite Levels in Vegetables Following Thermal Processing

While acknowledging that some of the analyzed vegetables are consumed after partial or complete thermal preparation—a process known to potentially alter nitrate levels according to specialized literature—our analysis was based on fresh, raw samples. This approach was chosen because these vegetables are predominantly consumed in this form, thus excluding the effect of thermal processing from our study. Consequently, for future investigations, it is essential to consider thermal treatments as a strategy for reducing nitrate content.

Washing and immersion of vegetable products before consumption can help reduce the nitrate content, due to their high solubility in water. Studies indicate that these practices can remove about 10–15% of nitrates, but the result may vary depending on the type of product, temperature and duration of the process [8]. Nitrate reduction varies greatly depending on the vegetable product, as it was found that leafy vegetables have a higher reduction rate than fruit and root and tuber vegetables.

Boiling of vegetable products leads to a decrease in nitrate content by 22–40% [69] as the chemical composition changes with effects on reducing the concentration of nitrates, due to weakening of the vegetal tissue that absorbs water, so nitrates become very soluble in water. The high solubility of nitrates accelerates this transfer, which explains the decrease in their concentration in the final product [70,71].

Also, steam cooking of certain vegetable products (carrots, zucchini, potatoes, cabbage) reduces the nitrate content between 3 and 34% [62,69,72]. The steam cooking process helps break down the cellular structure of vegetables, thus allowing for the gradual release of nitrates. This method has the advantage of better preserving the essential nutrients and texture of vegetables, making it a preferred option in some cases for food preparation.

If vegetable products are peeled, nitrate losses are higher [72,73]. The peel often retains a significant amount of nitrates, and its removal allows the direct removal of part of these compounds, thereby contributing to a decrease in the total concentration in the final food.

Regarding the frying of vegetable products, the nitrate content has an apparent increase between 12.46 and 29.93% [34] as the weight of the vegetable product by frying is reduced, which leads to a concentration of nitrates on a lower product weight. During frying, a significant amount of water is lost, which causes nitrates to be concentrated in a smaller volume. Thus, although no additional nitrates are added, their relative concentration increases due to dehydration of the plant tissue. This change is more a consequence of water loss than an absolute increase in nitrates.

Studies have shown that there is a decrease in nitrate content during the storage of vegetable products at an ambient temperature of 22–30 °C due to the denitrification process, which is very active at this temperature, followed by an increase in the nitrite content in these products [30,74,75,76]. In order to avoid this, it is recommended to store vegetable products at temperatures between 4 and 15 °C [77].

### 3.4. Dietary Exposure and Health Risk Assessment of Nitrate and Nitrite

The primary source of nitrates in human nutrition is the consumption of vegetables and cultivated plants (approximately 80%), especially leafy vegetables such as spinach, lettuce, and radishes. These plants accumulate nitrates naturally, particularly when nitrogen-rich fertilizers are applied to the soil. Drinking water contributes about 10% of total nitrate intake, particularly in regions affected by agricultural runoff or nitrate contamination. The remaining 10% originates from other food products, including processed meats and dairy.

**Figure 5:**
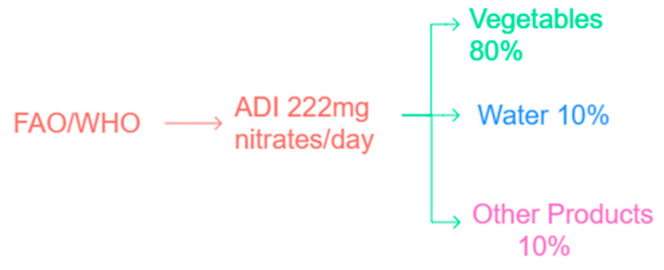
Estimated sources of daily nitrate intake relative to the Acceptable Daily Intake (ADI) recommended by FAO/WHO (222 mg/day).

Health agencies such as FAO/WHO and EFSA have established an Acceptable Daily Intake (ADI) for nitrate of 3.7 mg/kg body weight/day, which corresponds to approximately 222 mg/day for a 60 kg adult. This value represents a safe threshold for total daily exposure, not a permissible limit for individual food items. Figure 5 illustrates the estimated contribution of various sources to total nitrate intake in the general population.

The European Food Safety Authority (EFSA) has evaluated the safety of nitrate and nitrite intake and established Acceptable Daily Intakes (ADIs) to guide risk assessment. According to the EFSA Scientific Opinion published in 2017, the ADI for nitrates is set at 3.7 mg/kg body weight/day, expressed as nitrate (${NO}_{3}^{-}$), equivalent to 222 mg ${NO}_{3}^{-}$/day for an adult of 60 kg and to 99.9 mg ${NO}_{3}^{-}$ for a 27 kg child, while the ADI for nitrites is 0.07 mg/kg body weight/day, expressed as nitrite (${NO}_{2}^{-}$), equivalent to 4.2 mg ${NO}_{2}^{-}$/day for an adult of 60 kg and to 1.89 mg ${NO}_{2}^{-}$/day for a 27 kg child. These values align closely with those established by the Joint FAO/WHO Expert Committee on Food Additives (JECFA) [18].

**Figure 6:**
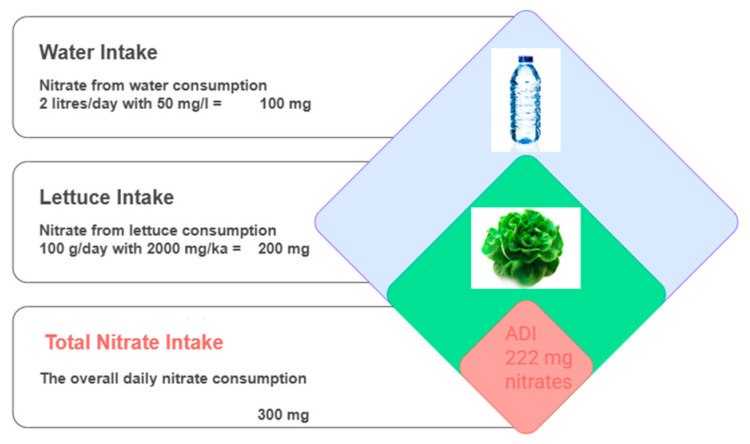
Daily nitrate intake in a human body (hypothetical example).

Acceptable daily intakes of 222 mg ${NO}_{3}^{-}$ established by FAO/WHO can be easily exceeded even if vegetable products have a ${NO}_{3}^{-}$ content below the maximum permissible limit for an adult. For example, if a person consumes only 100 g of lettuce per day, which has a nitrate load of 2000 mg/kg (the maximum permissible limit for lettuce in Romania), this amounts to 200 mg of nitrates ingested from the lettuce. Additionally, if the person consumes 2 L of water per day, as recommended, with a concentration of 50 mg/L, this would add another 100 mg of nitrates. In total, just from water and lettuce alone, the average person ingests 300 mg of nitrates per day, thereby exceeding the ADI of 222 mg/day (Figure 6).

Following nitrate ingestion, such as through the consumption of vegetables, approximately 25% of the ingested nitrate is secreted into the oral cavity via salivary secretion. Within the saliva, an estimated 20% of this nitrate undergoes reduction to nitrite mediated by commensal bacteria residing on the dorsal surface of the tongue. Consequently, in a healthy individual, approximately 5–7% of the total dietary nitrate intake is ultimately converted into nitrite within the oral cavity [78]. This biochemical transformation holds significant physiological relevance, as nitrite functions in various biological processes, including the modulation of vascular tone and antimicrobial activity. Nonetheless, elevated levels of nitrite pose potential health risks, which are contingent upon the concentration and contextual factors.

Assuming a person consumes 220 mg of nitrate, 5% of that is converted to nitrite in the saliva and this implies that 5% of 220 mg represents 11 mg of nitrite. Although a small proportion of ingested nitrate is endogenously converted into nitrite (approximately 5%), this physiological process has already been taken into account in the risk assessments performed by health agencies. Therefore, the ADI values established for nitrate and nitrite are mutually consistent and protective of human health. But the ADI for nitrite is only 3.6 mg, which means that the converted amount (11 mg) exceeds the nitrite ADI by over 3 times [60].

Even if someone stays within the safe limit for nitrate, the conversion to nitrite in the body (specifically in the oral cavity) can lead to nitrite exposure beyond its safe limit. This suggests a potential flaw or limitation in evaluating ${NO}_{3}^{-}$ and ${NO}_{2}^{-}$ ADIs separately, since they are biologically linked.

The findings suggest that current nitrate ADI values may underestimate true nitrite exposure, highlighting a possible need for revisiting dietary guidelines or safety assessments.

Given that leafy vegetables are known nitrate accumulators, it is advisable to monitor their intake, especially in populations vulnerable to nitrate exposure (e.g., children or individuals with specific health conditions). Ensuring a balanced consumption across different vegetable types, including fruits, root vegetables, and leafy greens, can help limit nitrate exposure while maintaining nutritional quality [79]. Moreover, consuming organically grown vegetables, which typically contain lower nitrate levels, may be a safer alternative [30].

Studies conducted in Bangladesh [24] and Poland [80] reported lower EDI values for carrots and tomatoes compared to our study, while for cucumbers and potatoes, the EDI values were higher than those found in the present study.

Mehri [81] reported lower EDI values for onions, carrots, tomatoes, and lettuce compared to our findings. The higher EDI values observed in our study, compared to other studies, can be attributed to the fact that the mean nitrate concentrations in our samples were higher than the levels reported elsewhere.

Regarding nitrites, the EDI values in our study are also higher than those reported in the study conducted in Korea [50]. However, all EDI values in our research remain below the ADI established by EFSA (0.07 mg/kg body weight/day), indicating a low health risk associated with the consumption of the analyzed vegetables.

### 3.5. Limitations

This study has several methodological limitations. Although the analytical method employed complies with ISO and AOAC standards, its reliance on the phenoldisulfonic acid colorimetric technique limits both specificity and sensitivity compared to modern instrumental approaches. This method is susceptible to matrix interferences and may yield less accurate results. Future research should consider more advanced techniques such as ion chromatography (IC) or high-performance liquid chromatography (HPLC) to improve analytical precision and reduce uncertainty. Finally, while the results are representative of local produce, caution is advised when generalizing to broader populations, as agricultural practices and environmental conditions can vary significantly across regions.

## 4. Materials and Methods

### 4.1. Samples

During the year 2023, a comprehensive monitoring study was conducted to assess the presence of nitrates and nitrites in various vegetable species in Dolj County. The vegetable products were purchased from the vegetable markets of the city of Craiova, located in the south of Dolj County, with a population of 234,140. Dolj County has a temperate continental climate with very hot summers and moderate winters with thermal values of 11.5 °C, with average annual precipitation amounts of 600 mm, the duration of sunshine is 2200–2300 h/year, with a solar irradiance of 1450 kWh/m^2^.

These markets are supplied with vegetable products from producers operating in the main agricultural production areas from the communes of Dolj County, ensuring a representation of local vegetable products. A total of nine representative public markets from different districts of Craiova were selected for sampling. A 500 g sample of each vegetable product was obtained from the local market. The selection aimed to cover both small local producers and large-scale vegetable growers. The focus was on 15 vegetables known for their agricultural significance in the region; a number of 20 samples for each species and a total of 300 vegetable samples at commercial maturity were collected randomly.

The samples were collected between May and October 2023, covering the full harvest season for the studied vegetable species in the region, and ensuring temporal representativeness.

The species included in this study were grouped in three categories: fruit vegetables—tomato (*Solanum lycopersicum)*, cucumber (*Cucumis sativus*), eggplant (*Solanum melongena*), green bean (*Phaseolus vulgaris*), bell pepper (Capsicum annuum L.) and hot peppers (Capsicum anuum L.); root and tuber vegetables—carrots (*Daucus carota* L.), potatoes (*Solanum tuberosum* L.) and onions (*Allium cepa* L.); leafy vegetables—dill (*Anethum graveolens*), parsley (*Petroselinum crispum*), lovage (*Levisticum officinale*), lettuce (*Lactuca sativa*), cabbage (*Brassica oleracea* L.) and spinach (*Spinacia oleracea*).

The studied species were cultivated in the field by local producers who provide vegetable products to the markets of Craiova and belong to 8 botanical families, namely *Amaranthaceae*, *Asteraceae*, *Solanaceae*, *Liliaceae*, *Cruciferae*, *Umbeliferae*, *Leguminosae* and *Cucurbitaceae*.

Vegetable samples were analyzed fresh. In the first phase, damaged leaves, dust or soil were removed.

To ensure statistical validity and repeatability of the data, each vegetable sample was analyzed in triplicate (*n* = 3), and the reported values for nitrate and nitrite concentrations represent the mean. Immediately after collection, vegetable samples were placed in sterile plastic bags, labeled, and transported to the laboratory under refrigerated conditions (4 °C) to minimize metabolic changes. Samples were analyzed within 24 h of collection to prevent alteration of nitrate and nitrite levels. Precautions were taken to avoid prolonged exposure to light and air, which could affect nitrate and nitrite stability.

To assess the accuracy and precision of the spectrophotometric method used in this study, method validation was performed using standard addition and recovery tests. Vegetable matrices were spiked with known concentrations of nitrate and nitrite standards, and recovery rates were calculated. The recovery for nitrates ranged between 91.2 and 104.3%, and 88.7–101.5% for nitrites, confirming good method accuracy. Precision was evaluated by repeated analysis (*n* = 3) of both spiked and unspiked samples, with relative standard deviation (RSD) values below 5% for both analytes. Calibration curves were prepared using five concentration levels (1.5–100 mg/kg for nitrates and 0.650 mg/kg for nitrites), and linearity was confirmed with R^2^ values above 0.998 for both analytes. These validation data confirm that the method is both accurate and precise for the determination of nitrates and nitrites in vegetable matrices.

All reagents used in the analysis were of analytical grade. Phenoldisulfonic acid was purchased from Sigma-Aldrich (Merck, Darmstadt, Germany). Sodium hydroxide, calcium carbonate, hydrogen peroxide and acetic acid (glacial) were obtained from *Merck* (Darmstadt, Germany). Activated carbon was supplied by Carlo Erba (Milan, Italy). All mass determinations were performed using an analytical balance Mettler Toledo AG204 (Greifensee, Switzerland), and homogenization was carried out with a Heidolph Promax 2020 orbital shaker (Schwabach, Bavaria, Germany). Deionized water was produced with a Millipore Direct-Q 3UV-R system (Merck KGaA, Darmstadt, Germany). Spectrophotometric measurements were performed using a Jasco V-460 UV–Vis spectrophotometer (Tokyo, Japan).

### 4.2. Determination of Nitrates

The vegetable product was roughly chopped or grated, and then 10 g was weighed and ground in a porcelain mortar.

Next, 1 g of active carbon was added over the sample and mixed until a homogeneous mass was obtained. Active carbon was added during sample preparation to adsorb pigments and other interfering substances, improving the clarity of the extract, without affecting nitrate or nitrite recovery. Gradually, 200 mL of 2% acetic acid was added, homogenizing with the pestle for 3–5 min. The suspension was filtered, and 5 mL of the filtrate was pipetted into a porcelain capsule. Then, 1 mL of 30% perhydrol and 2 mL of 10% NaOH were added.

The contents of the capsule were evaporated to dryness. The sample was treated with 2 mL of phenoldisulfonic acid and then 25 mL of deionized water was added and neutralized with NaOH 40%, until the yellow coloration was formed. The contents of the capsule were placed in a 100 mL volumetric flask and brought to the mark with deionized water. Absorbance was measured with a Jasco-460 spectrophotometer at a wavelength of 420 nm.

(1) $$N-{\mathrm{NO}}_{3}\;(\mathrm{mg}/\mathrm{kg})=\frac{C\;\times \;V1}{V2\;\times \;m}$$

C: the N-NO_3_ content read on the calibration curve;

V1: total volume of extractive solution (mL);

V2: volume of extract used for dosing (mL);

m: mass of plant material (grams).

Each result of the analysis was compared with the maximum permissible limit, thus establishing whether or not there are vegetable products contaminated with nitrites and nitrites sold in the markets of the city of Craiova.

### 4.3. Determination of Nitrites

A 5 mL aliquot from the extract obtained for nitrate determination was pipetted into a porcelain capsule. Then, 1 mL of 30% perhydrol and 2 mL of CaCO_3_ were added. The contents of the capsule were evaporated to dryness. The sample was treated with 2 mL of phenoldisulfonic acid for 2–3 min and then 25 mL of deionized water was added and neutralized with NaOH 40% until it turned yellow. The contents of the capsule were passed into a 100 mL volumetric flask and was made up to the mark with deionized water. The samples were read with a Jasco-460 spectrophotometer at a wavelength of 520 nm.

(2) $${\mathrm{NO}}_{2}=\frac{C\;\times \;V1}{V2\;\times \;m}$$

C: the NO_2_ content read on the calibration curve;

V1: total volume of extractive solution (mL);

V2: volume of extract used for dosing (mL);

m: mass of plant material (grams).

The concentration of nitrate and nitrite in vegetable products is expressed in the form of mg/kg fresh weight (FW).

To ensure the accuracy and reliability of nitrate and nitrite determinations, the analytical procedures employed in this study were aligned with internationally recognized protocols. Specifically, the methodology used for nitrate and nitrite analysis was based on the colorimetric method adapted from the standard ISO 6635:1984 (Fruits, vegetables and derived products-Determination of nitrate content-Spectrometric method) [82] and the AOAC Official Method 973.31 for nitrate and nitrite in plant tissues. These methods are widely accepted for quantifying nitrate and nitrite contents in vegetables due to their sensitivity and reproducibility.

The limits of detection (LOD) and quantification (LOQ) were established to evaluate the reliability of the measurements, particularly at low concentration levels. For nitrates (NO_3_^−^), the LOD was 0.5 mg/kg and the LOQ was 1.5 mg/kg. For nitrites (NO_2_^−^), the LOD was 0.2 mg/kg and the LOQ was 0.6 mg/kg. These limits were determined based on signal-to-noise ratios of 3:1 for LOD and 10:1 for LOQ, respectively, using calibration curves prepared with certified reference standards. All measurements reported in this study were above the respective LOQ values.

### 4.4. Statistical Analysis

The statistical analysis was performed using Minitab version 22.1. Data distribution was evaluated with Kolmogorov–Smirnov, Anderson–Darling, and Ryan–Joiner tests, all indicating significant deviations from normality (*p* < 0.05). Consequently, non-parametric approaches were applied, with results summarized by medians, interquartile ranges, and extreme values. Boxplots were used to illustrate variability and outliers, while heatmaps based on mean concentration values provided comparative visualization among vegetable species. Given the lack of normality, parametric ANOVA tests were not applied.

To ensure analytical reliability and reproducibility, a quality assurance and quality control (QA/QC) protocol was applied throughout the experimental procedures. All vegetable samples were analyzed in triplicate, and the mean values were reported. Procedural blanks were included in each analytical batch to check for contamination, and calibration standards were freshly prepared and verified before each series of measurements. Where available, certified reference materials (CRMs) for nitrate and nitrite in plant-based matrices were used to verify method performance and assess accuracy. Additionally, instrument calibration was routinely verified using internal quality control samples. These QA/QC measures ensured consistent analytical performance and enhanced the credibility of the nitrate and nitrite quantifications.

### 4.5. Health Risk Assessment

The risk assessment methodology applied in this study is based on internationally recognized frameworks recommended by JECFA, EFSA, and the US EPA, and was used to estimate the daily intake of nitrates and nitrites and to evaluate potential health risks by comparing the estimated intake to established reference doses [16,25,35,83].

Daily intake for nitrates and nitrites (expressed in mg/day) was calculated, taking into account the annual consumption of vegetable products (kg/year) mentioned in Table 13, which was multiplied by the mean value of ${NO}_{3}^{-}/or\;{NO}_{2}^{-}$ (mg/kg) in that type of vegetable, and divided by the number of days in a year, i.e., 365.

Table 13 shows the average annual consumption of vegetables per inhabitant based on consumption availability of the population in 2022, published in 2024 by National Institute of Statistics [17].

EDI was calculated for two groups of the population (children and adults) taking into account the average daily consumption of vegetables, which according to the NIS [17] is 246.5 kg/inhabitant/year, as well as the minimum consumption of 400 g of fruit and vegetable per individual/day recommended by FAO/WHO for the European population, meaning 146 kg/year [84].

The estimated daily intake (EDI) was calculated with Equation (3) [24].

EDI = (C × IR)/BW (3)

where C: concentration of nitrates or nitrites (mg/kg);

IR: intake rate (mg/day);

BW: body weight (kg).

The Health Risk Index (HRI) for nitrates/nitrites was calculated in the same way as EDI, for both children and adults, taking into account both the daily consumption of vegetables in Romania in 2022, but also the minimum consumption recommended at European level, which is 400 g of fruit and vegetables, using Equation (4).

(4) $$\mathrm{HRI}=\frac{EDI}{Rfd}$$

where *EDI* is Estimated Daily Intake (mg/kg body weight/day),

*Rfd* is the reference dose (mg/kg body weight/day).

In this dietary risk assessment, body weights of 60 kg for adults and 27 kg for children were used. These values are consistent with standard assumptions historically used in international risk assessments and serve to ensure comparability with prior evaluations.

The adult reference weight of 60 kg has been widely employed in toxicological assessments by international bodies such as the Joint FAO/WHO Expert Committee on Food Additives (JECFA), even though it is not explicitly stated in their 2002 evaluation. This weight represents a generalized global average used for modeling exposures in adult populations, particularly in contexts where population-specific anthropometric data are not available. For example, using a 60 kg adult, the JECFA ADI of 3.7 mg nitrate/kg bw/day translates to 222 mg nitrate/day, an often cited value in both regulatory documents and academic literature [57].

Similarly, the 27 kg reference weight for children reflects a typical body weight for a child aged approximately 6 to 8 years. This assumption aligns with standard practice in exposure assessment when age-stratified growth data are not being applied. It provides a conservative estimate of exposure in a vulnerable subpopulation (children), who are generally more sensitive to contaminants due to their higher intake-to-body weight ratios.

The assessment of non-carcinogenic risk associated with nitrate and nitrite ingestion through vegetable consumption is a crucial component of dietary exposure analysis. In this study, the applied methodology, in accordance with international guidelines, enables risk quantification via the Health Risk Index (HRI). According to international consensus, HRI values below 1 indicate a negligible risk to human health, whereas HRI values exceeding 1 suggest a potential non-carcinogenic risk, highlighting the need for precautionary measures [83].

Chronic exposure to nitrates and nitrites may lead to several non-carcinogenic health effects, including methemoglobinemia, a condition characterized by the oxidation of hemoglobin to methemoglobin reducing oxygen transport capacity [8,85], which is particularly hazardous in infants and young children [16]; disruption of thyroid function through competitive inhibition of iodine uptake, potentially contributing to goiter and hypothyroidism [86]; and alterations in blood pressure and other cardiovascular effects, observed under conditions of prolonged and elevated exposure [8,87,88]. The occurrence of HRI values > 1 in certain frequently consumed vegetables highlights the need for targeted intervention, particularly to safeguard vulnerable groups such as children, pregnant women, and individuals with pre-existing health conditions. In this regard, several measures are recommended:

- Systematic monitoring of nitrate concentrations in vegetable products, especially in agricultural areas with intensive fertilization practices;
- Implementation of sustainable agricultural policies and best practice guidelines for farmers to limit the excessive use of nitrogen-based fertilizers;
- Promotion of public awareness initiatives to encourage responsible consumption of vegetables with a high potential for nitrate accumulation;
- Encouragement of dietary diversification to reduce continuous exposure to nitrate-rich vegetables and to increase the consumption of low-nitrate alternatives.

The oral reference dose (Rfd) is 7.09 mg ${NO}_{3}^{-}$/kg bw/day for nitrate and 0.33 mg ${NO}_{2}^{-}$/kg bw/day for nitrite, representing the estimated daily exposure considered unlikely to cause adverse non-carcinogenic health risks [89].

The recorded values for the estimated daily intake were compared with the acceptable daily intake (ADI) to establish whether the consumption of vegetables containing nitrates represents a potential risk for human health.

## 5. Conclusions

Recent studies highlight the importance of regulating the nitrate content of vegetables, given the impact on human health. Legal limits for nitrates in vegetables are essential for the protection of public health, and the variability of nitrate content depending on the type of vegetable and growing conditions is significant. Also, the health risks associated with the consumption of vegetables with nitrates highlight the need for strict control and regulatory measures.

Studying the content of nitrates and nitrite in vegetable products sold in the agro-food markets in Craiova by local producers represents a very important objective in terms of the health of the city’s population.

For this reason, we studied the concentration of nitrates and nitrite in various vegetable products produced in the city’s neighboring areas and sold in markets and found that 1.66% of the vegetable products studied (300 samples with 15 species) exceed the maximum permissible limit for nitrates, that is, 5 samples (2 tomato samples, 2 eggplant samples and 1 bell pepper sample). Regarding the nitrite content, it exceeds the maximum permissible limit in 7.66% of the analyzed samples. Although the percentage is small, it must be taken into account that nitrites are much more toxic than nitrates, as they can form nitrosamines, substances with a mutagenic effect and which lead to the appearance of various forms of cancer through accumulation.

The HRI values in particular for children consuming lettuce and lovage exceed 1 (1.14 and 1.65, respectively), clearly indicating a potential non-carcinogenic health risk associated with regular consumption. This finding highlights the need for consumption moderation, especially among vulnerable groups such as children. For adults, the HRI values remain below 1, suggesting no immediate risk, although high EDIs in some leafy vegetables like lovage warrant attention.

If we compare HRI exposure to nitrates and nitrite according to FAO/WHO recommendation of consumption of 400 g/day, we observe that only in the case of children and only in the lettuce and lovage samples, HRI presents a risk of exposure to nitrates.

Taking into account the results obtained in this study, we can consider the consumption of vegetable products from the city markets to be safe with regard to the intake of nitrates and nitrite.

It must be taken into account that not only vegetable products are sources of nitrates and nitrite for the human body. In addition to these, other sources are represented by water and food products, which should be studied together to finally determine the health risk.

The present study primarily focused on the quantification and health risk assessment of nitrate and nitrite content in vegetables sold in Craiova markets, but it also considered the context of regional agricultural practices that may influence these levels. Given the temperate–continental climate of Dolj County, with high solar irradiance and long sunshine duration, early maturation of vegetable crops is common and may influence the uptake and accumulation of nitrogen compounds. Control measures for reducing nitrate and nitrite accumulation, such as optimized nitrogen fertilization, appropriate irrigation schedules, crop rotation, and the use of cultivars with low nitrate accumulation potential, were reviewed as part of the broader interpretation of the results.

However, beyond mentioning these practices, the feasibility and effectiveness of specific control strategies were also considered. In the context of Dolj County, where vegetable cultivation is often carried out by small- and medium-scale farmers, the application of organic fertilizers (e.g., manure, compost) and precision fertilization techniques remains a realistic approach. These methods are accessible to local producers and have been documented in the literature as effective in reducing nitrate accumulation in leafy and root vegetables. Studies have shown, for example, that reducing excessive nitrogen input by 30–40% without compromising yield can significantly decrease nitrate content in spinach and lettuce [7].

Furthermore, considering that vegetable samples collected in this study originated from multiple local producers and varied soil types, the implementation of customized fertilization protocols adapted to soil characteristics and crop species may enhance the effectiveness of control measures. Although a systematic field intervention was not part of the current study design, the findings provide a basis for future applied research focused on evaluating the practical outcomes of such interventions under local agronomic conditions.

The results obtained in this study provide a scientific basis for supporting local and national authorities in the development of specific control measures and maximum allowable limits adapted to local agricultural conditions. The relatively low health risk identified for most of the vegetable samples suggests that current agricultural practices in the Dolj region are generally safe; however, the presence of samples exceeding legal thresholds underlines the need to strengthen monitoring systems, particularly in high-risk crops such as leafy and fruiting vegetables. These findings may serve as reference data for adjusting fertilizer management strategies at the farm level, especially in the context of small-scale producers who contribute significantly to regional vegetable supply.

From the perspective of consumer guidance, the study emphasizes the importance of informed choices when purchasing vegetables, particularly for sensitive groups such as children. Public health authorities could consider disseminating clear recommendations on washing, storing, and cooking vegetables to reduce nitrate and nitrite intake, as well as promoting consumption of species with consistently low levels.

This study presents relevant data on the levels of nitrate and nitrite in vegetables cultivated in Dolj County, Romania, and evaluates the potential health risks associated with their dietary intake. The findings contribute to a better understanding of the presence of these compounds in commonly consumed vegetables and highlight the importance of continued monitoring for consumer safety.

Future research should address the temporal variability of nitrate and nitrite concentrations across different seasons and cultivation systems (e.g., conventional vs. organic farming). In addition, the influence of agronomic factors, such as fertilization practices, irrigation regimes, and soil characteristics, warrants further investigation. Comprehensive exposure assessments that include multiple dietary sources, such as vegetables, drinking water, and processed foods, are also essential to better characterize potential health risks.

These directions may guide the development of more effective strategies and policies to minimize nitrate and nitrite exposure through diet and support informed risk management decisions.

## Figures and Tables

**Table 1:** Nitrate content in fruit vegetables.

Fruit Vegetables	Concentration of ${NO}_{3}^{-}$ (mg/kg FW)			The MPL of Nitrate * (mg/kg FW)
	Mean ± SD	Median	Concentration Range	
Tomato	74.2 ± 36.5	68.0	34.0–179.0	150
Cucumber	53.2 ± 16.5	49.5	23.0–98.0	200
Eggplant	145.3 ± 179.9	95.0	45.0–870.0	300
Green bean	122.9 ± 70.6	98.0	50.0–320.0	-
Bell pepper	96.4 ± 82.5	72.5	24.0–420.0	150
Hot pepper	78.15 ± 29.7	73.0	39.0–140.0	150

* According to Order no. 1/2002 [13]. FW: fresh weight, SD: standard deviation; MPL: maximum permissible limit.

**Table 2:** Nitrate content in leafy vegetables.

Leafy Vegetables	Concentration of ${NO}_{3}^{-}$ (mg/kg FW)			The MPL of Nitrate (mg/kg FW)
	Mean ± SD	Median	Concentration Range	
Lettuce	547.7 ± 386.0	526.5	87.0–1223.0	2000 *; 3000 **
Cabbage	157.2 ± 99.4	121.0	25.0–378.0	-
Spinach	294.3 ± 113.4	265.5	137.0–497.0	2000 *; 3500 **
Dill	364 ± 138.0	337.5	199.0–630.0	-
Parsley	245.9 ± 82.8	221.5	134.0–445.0	-
Lovage	794.35 ± 139.9	832.0	440.0–970.0	-

* According to Order no. 1/2002 [13]. ** According to Commission Regulation (EU) No. 915/2023 [14]. FW: fresh weight, SD: standard deviation; MPL: maximum permissible limit.

**Table 3:** Nitrate content in root and tuber vegetables.

Root and Tuber Vegetables	Concentration of ${NO}_{3}^{-}$ (mg/kg FW)			The MPL of Nitrate * (mg/kg FW)
	Mean ± SD	Median	Concentration Range	
Carrots	95.6 ± 28.6	91.0	53.0–149.0	400
Potatoes	108.8 ± 36.0	98.0	68.0–210.0	300
Onions	72.8 ± 34.2	65.0	30.0–153.0	200

* According to Order no. 1/2002 [13]. FW: fresh weight, SD: standard deviation; MPL: maximum permissible limit.

**Table 4:** Nitrite content in fruit vegetables.

Fruit Vegetables	Concentration of ${NO}_{2}^{-}$ (mg/kg FW)			The MPL of Nitrite * (mg/kg FW)
	Mean ± SD	Median	Concentration Range	
Tomato	0.49 ± 0.17	0.50	0.20–0.90	1
Cucumber	0.54 ± 0.11	0.50	0.40–0.80	
Eggplant	0.78 ± 0.35	0.70	0.40–1.90	
Green bean	0.62 ± 0.29	0.50	0.20–1.20	
Bell pepper	0.49 ± 0.14	0.50	0.20–0.80	
Hot pepper	0.49 ± 0.14	0.50	0.20–0.80	

* According to Order no. 1/2002 [13]. FW: fresh weight, SD: standard deviation; MPL: maximum permissible limit.

**Table 5:** Nitrite content in leafy vegetables.

Leafy Vegetables	Concentration of ${NO}_{2}^{-}$ (mg/kg FW)			The MPL of Nitrite * (mg/kg FW)
	Mean ± SD	Median	Concentration Range	
Lettuce	0.79 ± 0.42	0.75	0.20–1.70	1
Cabbage	0.67 ± 0.31	0.65	0.10–1.10	
Spinach	0.94 ± 0.37	0.85	0.50–1.60	
Dill	0.61 ± 0.16	0.60	0.40–1.10	
Parsley	0.57 ± 0.20	0.60	0.20–0.90	
Lovage	0.74 ± 0.13	0.70	0.50–1.10	

* According to Order no. 1/2002 [13]. FW: fresh weight, SD: standard deviation; MPL: maximum permissible limit.

**Table 6:** Nitrite content in root and tuber vegetables.

Root and Tuber Vegetables	Concentration of ${NO}_{2}^{-}$ (mg/kg FW)			The MPL of Nitrite * (mg/kg FW)
	Mean ± SD	Median	Concentration Range	
Carrots	0.60 ± 0.20	0.65	0.30–0.90	1
Potatoes	0.65 ± 0.17	0.60	0.40–0.90	
Onions	0.60 ± 0.19	0.60	0.30–0.90	

* According to Order no. 1/2002 [13]. FW: fresh weight, SD: standard deviation; MPL: maximum permissible limit.

**Table 7:** Health risk exposure of nitrate and nitrite according to daily intake.

Vegetable Samples		Nitrate							Nitrite					
	DI (mg/day)	EDI		% ADI		HRI		DI (mg/day)	EDI		% ADI		HRI	
		Children	Adult	Children	Adult	Children	Adult		Children	Adult	Children	Adult	Children	Adult
**Fruit vegetables**														
Tomato	7.39	0.67	0.30	18.10	8.10	0.09	0.04	0.04	0.004	0.002	5.71	2.85	0.012	0.006
Cucumber	1.12	0.48	0.21	12.97	5.67	0.06	0.02	0.01	0.004	0.002	5.71	2.85	0.012	0.006
Eggplant	-	1.32	0.59	35.67	15.9	0.18	0.08	-	0.007	0.003	10.00	4.28	0.021	0.009
Green bean	0.94	1.11	0.50	30.00	13.51	0.15	0.07	<0.01	0.005	0.002	7.14	2.85	0.015	0.006
Bell pepper	3.27	0.87	0.39	23.51	10.54	0.20	0.05	0.01	0.004	0.002	5.71	2.85	0.012	0.006
Hot pepper	-	0.72	0.32	19.45	8.64	0.10	0.04	-	0.004	0.002	5.71	2.85	0.012	0.006
**Leafy vegetables**														
Lettuce	-	4.99	2.24	134.86	60.54	0.70	0.31	-	0.007	0.003	10.00	4.28	0.021	0.012
Cabbage	19.42	0.66	0.29	17.83	7.83	0.09	0.04	0.08	0.006	0.002	8.57	2.85	0.018	0.006
Spinach	-	2.68	1.20	72.43	32.43	0.37	0.16	-	0.008	0.003	11.42	4.25	0.024	0.009
Dill	-	3.31	1.49	89.45	40.27	0.46	0.21	-	0.005	0.002	7.14	5.71	0.015	0.006
Parsley	-	2.24	1.00	60.54	0.27	0.31	0.14	-	0.005	0.002	7.14	2.85	0.015	0.006
Lovage	-	7.23	3.25	195.40	87.83	1.01	0.45	-	0.006	0.003	8.57	4.25	0.018	0.009
**Root and tuber vegetables**														
Carrots	-	0.87	0.39	0.23	10.54	0.12	0.05	-	0.005	0.002	7.14	5.71	0.015	0.006
Potatoes	29.1	0.99	0.44	0.26	11.89	0.13	0.06	0.17	0.005	0.002	7.14	2.85	0.015	0.006
Onions	3.51	0.66	0.29	17.83	7.83	0.09	0.04	0.02	0.005	0.002	7.14	2.85	0.015	0.006

DI: Daily intake nitrate/nitrite (mg/day) values are based on national availability data from the National Institute of Statistics (2024) and may overestimate real intake, as they include potential food waste and inedible portions. The same average intake values were used for both children and adults due to the lack of age-disaggregated data; EDI: estimated daily intake (mg/kg body weight/day), it is calculated according to Equation (3). EDI was calculated based on average body weights of 60 kg for adults and 27 kg for children; ADI: acceptable daily intake, 3.7 mg/kg bw/day for nitrate, 0.07 mg/kg bw/day for nitrite; HRI: Health Risk Index calculated according to Equation (4); Rfd: oral reference dose (mg/kg body weight/day). For nitrates, it is 7.09 mg ${NO}_{3}^{-}$/kg bw/day and for nitrite, it is 0.33 mg ${NO}_{2}^{-}$/kg bw/day.

**Table 8:** Health risk exposure of nitrates and nitrite according to FAO/WHO recommendation of consumption of 400 g/day.

Vegetable Samples	Nitrate						Nitrite					
	EDI		% ADI		HRI		EDI		% ADI		HRI	
	Children	Adult	Children	Adult	Children	Adult	Children	Adult	Children	Adult	Children	Adult
**Fruit vegetables**												
Tomato	1.09	0.49	29.45	13.24	0.15	0.06	0.007	0.003	10.00	4.28	0.021	0.009
Cucumber	0.78	0.35	21.08	9.45	0.11	0.04	0.008	0.003	11.42	4.28	0.024	0.009
Eggplant	2.15	0.96	58.10	25.94	0.30	0.05	0.011	0.005	15.71	7.14	0.033	0.015
Green bean	1.82	0.81	49.18	21.89	0.25	0.11	0.009	0.004	12.85	5.71	0.027	0.012
Bell pepper	1.42	0.64	38.37	17.29	0.20	0.09	0.007	0.003	10.00	4.28	0.015	0.009
Hot pepper	1.15	0.52	31.08	14.05	0.16	0.07	0.007	0.003	10.00	4.28	0.021	0.009
**Leafy vegetables**												
Lettuce	8.11	3.65	219.18	98.64	1.14	0.51	0.011	0.005	15.71	7.14	0.033	0.015
Cabbage	1.09	0.48	29.45	12.97	0.15	0.06	0.009	0.004	12.85	5.71	0.027	0.012
Spinach	4.36	1.96	117.83	52.97	0.61	0.27	0.013	0.006	18.57	8.57	0.039	0.018
Dill	5.39	2.42	145.67	65.40	0.76	0.34	0.009	0.004	12.85	5.71	0.027	0.012
Parsley	3.64	1.63	98.37	44.05	0.51	0.22	0.008	0.003	11.42	4.25	0.024	0.009
Lovage	11.76	5.29	317.83	142.97	1.65	0.74	0.010	0.004	14.28	5.71	0.030	0.012
**Root and tuber vegetables**												
Carrots	1.41	0.63	38.10	17.02	0.19	0.08	0.008	0.004	11.42	5.71	0.024	0.012
Potatoes	1.61	0.72	43.51	19.45	0.22	0.10	0.009	0.004	12.85	5.71	0.027	0.012
Onions	1.07	0.48	28.91	12.97	0.15	0.06	0.008	0.004	11.42	5.71	0.024	0.012

EDI: estimated daily intake (mg/kg body weight/day), calculated according to Equation (3), using average body weights of 60 kg for adults and 27 kg for children; %ADI: percentage of the Acceptable Daily Intake established by FAO/WHO (2002)—3.7 mg/kg bw/day for nitrate and 0.07 mg/kg bw/day for nitrite; HRI: Health Risk Index, calculated as EDI/RfD, where the oral reference dose (RfD) is 7.09 mg/kg bw/day for nitrate and 0.33 mg/kg bw/day for nitrite (U.S. EPA IRIS, 1991). These values differ from the FAO/WHO ADI (3.7 and 0.07 mg/kg bw/day, respectively) and from the ATSDR Minimal Risk Levels (4.0 and 0.1 mg/kg bw/day, respectively), but were selected as protective benchmarks; FAO/WHO recommendation: the 400 g/day reference intake refers to the combined daily intake of fruits and vegetables for adults. In this study, it was conservatively applied to vegetables only and uniformly for both adults and children, which may lead to overestimation of exposure in children and unrealistic scenarios for some herbs (e.g., lovage or dill). The results should therefore be interpreted as worst-case exposure estimates.

**Table 9:** Classification of vegetable products according to nitrate content.

Nitrate Content (mg/kg FW)	Vegetable
Very low < 200	artichoke, asparagus, broad bean, brussels sprouts, eggplant, garlic, onion, green bean, melon, mushroom, pea, pepper, potato, summer squash, sweet potato, tomato, watermelon
Low 200–500	broccoli, carrot, cauliflower, cucumber, pumpkin, “puntarelle” chicory
Middle 500–1000	cabbage, “cima di rapa” (broccoli raab), dill, “radicchio”, savoy cabbage, turnip
High 1000–2500	celeriac, chinese cabbage, endive, escarola, fennel, kohlrabi, leaf chicory, leek, parsley
Very high > 2500	celery, chervil, cress, lamb’s lettuce, lettuce, radish, red beetroot, rocket (rucola), spinach, Swiss chard

(Source: Santamaria [7]). FW: Fresh weight.

**Table 10:** Classification of vegetable products into classes according to nitrate concentration.

Vegetables	Concentration Range	Classes of Nitrate Concentration (mg/kg)									
		Class I Very Low, <200		Class II Low, 200–500		Class III Middle, 500–1000		Class IV High, 1000–2500		Class V Very High, >2500	
		No. of Samples	%	No. of Samples	%	No. of Samples	%	No. of Samples	%	No. of Samples	%
**Fruit vegetables**											
Tomato	34–179	20	16.67	-	-	-	-	-	-	-	-
Cucumber	23–98	20	16.67	-	-	-	-	-	-	-	-
Eggplant	45–870	18	15	1	0.83	1	0.83	-	-	-	-
Green bean	50–320	17	14.17	3	2.5	-	-	-	-	-	-
Bell pepper	24–420	19	15.83	1	0.83	-	-	-	-	-	-
Hot pepper	39–140	20	16.67	-	-	-	-	-	-	-	-
Total category *n* = 120		114	95.01	5	4.16	1	0.83	-	-	-	-
Total vegetables, *n* = 300		114	38	5	1.67	1	0.33	-	-	-	
**Leafy vegetables**											
Lettuce	87–1223	7	5.83	2	1.67	9	7.5	2	1.67	-	-
Cabbage	25–378	15	12.5	5	4.16	-	-	-	-	-	-
Spinach	137–497	4	3.33	16	13.33	-	-	-	-	-	-
Dill	199–630	1	0.83	15	12.5	4	3.33	-	-	-	-
Parsley	134–445	8	6.67	12	10	-	-	-	-	-	-
Lovage	440–970	-	-	2	1.67	18	15	-	-	-	-
Total category *n* = 120		35	29.16	52	43.33	31	25.83	2	1.67	-	-
Total vegetables, *n* = 300		35	11.67	52	17.33	31	10.33	2	0.67	-	-
**Root and tuber vegetables**											
Carrots	53–149	20	33.33	-	-	-	-	-	-	-	-
Potatoes	68–210	19	31.67	1	1.67	-	-	-	-	-	-
Onions	30–153	20	33.33	-	-	-	-	-	-	-	-
Total category *n* = 60		59	98.33	1	1.67	-	-	-	-	-	-
Total vegetables, *n* = 300		59	19.67	1	0.33	-	-	-	-	-	-

*n*: number of samples.

**Table 11:** The classification of nitrate accumulation in fresh vegetables.

Levels	${NO}_{3}^{-}$ (mg/kg FW)	Contamination Degree	Dietary Limits
I	≤432	Slightly contaminated	Can be eaten raw
II	432–785	Moderately contaminated	Cannot be eaten raw, but can be pickled and cooked
III	785–1440	Heavily contaminated	Cannot be eaten raw or pickled, but can be cooked
IV	1440–3100	Critically contaminated	Cannot be eaten raw, pickled, or cooked, but is not poisonous

(Source: Shen, 1982 [15]). FW: Fresh weight.

**Table 12:** Classification of vegetable products into levels depending on nitrate concentration.

Vegetables	Concentration Range	Levels of Nitrate Concentration (mg/kg)							
		I, ≤432		II, 432–785		III, 785–1440		IV, 1440–3100	
		No. of Samples	%	No. of Samples	%	No. of Sample	%	No. of Samples	%
**Fruit vegetables**									
Tomato	34–179	20	16.67	-	-	-	-	-	-
Cucumber	23–98	20	16.67	-	-	-	-	-	-
Eggplant	45–870	19	15.82	-	-	1	0.83	-	-
Green bean	50–320	20	16.67	-	-	-	-	-	-
Bell pepper	24–420	20	16.67	-	-	-	-	-	-
Hot pepper	39–140	20	16.67	-	-	-	-	-	-
Total category *n* = 120		119	99.17	-	-	1	0.83	-	-
Total vegetables, *n* = 300		119	39.67	-	-	1	0.33	-	-
**Leafy vegetables**									
Lettuce	87–1223	9	7.5	3	2.5	8	6.67	-	-
Cabbage	25–378	20	16.67	-	-	-	-	-	-
Spinach	137–497	16	13.33	4	3.33	-	-	-	-
Dill	199–630	14	11.67	6	5	-	-	-	-
Parsley	134–445	19	15.83	1	0.83	-	-	-	-
Lovage	440–970	-	-	5	4.17	15	12.5	-	-
Total category, *n* = 120		78	65	19	15.83	23	19.17	-	-
Total vegetables, *n* = 300		78	26	19	6.33	23	7.67	-	-
**Root and tuber vegetables**									
Carrots	53–149	20	33.33	-	-	-	-	-	-
Potatoes	68–210	20	33.33	-	-	-	-	-	-
Onions	30–153	20	33.34	-	-	-	-	-	-
Total category *n* = 60		60	100	-	-	-	-	-	
Total vegetables, *n* = 300		60	20	-	-	-	-	-	-

*n*: number of samples.

**Table 13:** Annual consumption of vegetables per inhabitant in Romania.

Species	Annual Consumption of Vegetables (kg/Inhabitant)/Year
Tomato	36.4
Cucumber	7.7
Green bean	2.8
Bell pepper	12.4
Cabbage	45.1
Potatoes	97.7
Onions	17.6

## Data Availability

The original contributions presented in this study are included in the article. Further inquiries can be directed to the corresponding author.
